# Transmantle heterotopia associated with agenesis of the corpus callosum in a patient with Parkinson: A case report

**DOI:** 10.1016/j.radcr.2025.02.034

**Published:** 2025-03-08

**Authors:** Mattia Russel Pantalone, Stanislav Beniaminov, Daniel Martin Munoz, Francesca De Luca

**Affiliations:** a Department of Neurosurgery, Karolinska University Hospital, Stockholm, Sweden; b Department of Medicine, Karolinska Institute, Stockholm, Sweden; c Department of Neurology, Södersjukhuset, Stockholm, Sweden; d Department of Neuroradiology, Karolinska University Hospital, Stockholm, Sweden; e Department of Clinical Neuroscience, Karolinska Institute, Stockholm, Sweden

**Keywords:** Parkinson disease, Agenesis of corpus callosum, Transmantle heterotopia

## Abstract

Congenital malformations of the brain that can manifest at different ages with a wide spectrum of neurological symptoms. Although they have been described in adults, their relevance in the elderly in relation to other neurodegenerative disease is not well defined. Here we described the case of a man with the agenesis of the corpus callosum and transmantle heterotopia being diagnosed with Parkinson and we analyze the diagnostic challenges this case presented.

## Introduction

We present here the case of a 66 year old. Caucasian man referred by a family medicine doctor to the outpatient neurological clinic at Karolinska University Hospital in April 2023 with gait disturbances, back pain, and cognitive deterioration that had progressed in the previous 5 years. The patient was a retired taxi driver with a medical history of former smoking, moderate alcohol consumption, surgery for a bulging disc in the lumbar spine in the 90 s, ischemic heart disease, hypertension, hyperlipidemia, hereditary hemochromatosis with ongoing apheresis treatment, and minimal change glomerulopathy in remission.

Medications at the time of the first visit included acetylsalicylic acid, omeprazole, isosorbide mononitrate, atorvastatin, bisoprolol, chlorzoxazone, paracetamol, nitroglycerin spray. The patient´s father had been diagnosed with Parkinson's disease. No other neurological diseases were known in family history.

### Current medical history

The patient had always been physically active, and according to him, his main symptom was pain in the lower back, which had become significantly worse after he played intensive golf in the autumn of 2021. His gait and balance had also gotten worse over time. He had received NSAID via a primary care physician and had been to a physiotherapist without symptomatic improvement. A magnetic resonance imaging (MRI) of the lumbar spine showed disc bulging at multiple levels with associated foraminal stenosis at the level L4-S1, and the spine surgeon had already recommended conservative treatment. According to his wife, the patient had also deteriorated in memory over time, and cognition had become quieter and less active in recent years. For that, he underwent a cognitive test in 2019, and 2 brain CT scans in 2018 and 2019, revealing GCA grade 1, MTA grade 2 bilaterally, no white matter lesions (Fazekas grade 0), and agenesis of the corpus callosum (ACC). No specific radiological signs for dementia were diagnosed at the time.

### Neurological examination

The neurological examination at our hospital in 2023 revealed normal cognitive function and an appropriate fund of knowledge. No cranial nerve impairment was observed, normal facial motor skills, and no hypomimia. Grip strength was adequate bilaterally, but fine motor skills were impaired in the left hand, with reduced amplitude when moving the thumb and index finger. Normal grasset was found with good strength in both legs. No gear phenomena were observed in any of the extremities, although the foot tapping test showed reduced movement amplitude and slight start-up difficulties. The patient's gait was characterized by short steps and forward head posture. Impaired balance was observed, as the patient could not follow a straight line and showed a tendency to fall backward at the Romberg test. The patient experienced back pain while walking and lifting both legs, especially on the left side. Nontriggerable reflexes were found bilaterally in the lower extremities and no difficult-to-trigger reflexes in the arms. Babinski was negative.

### Clinical management

Given the patient's neurological status, the neurological consultation at our hospital posed clinical suspicion of Parkinson's disease. The patient was offered Levodopa treatment based on his clinical symptoms, but he preferred to await further radiological investigation before initiating medical therapy.

**Fig. 1:**
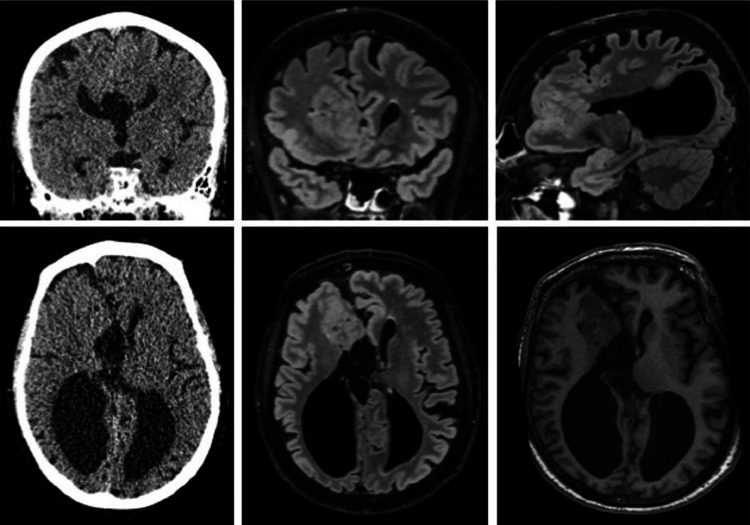
Brain CT from 2019, soft-tissue coronal and axial images (top right and bottom right); Brain MRI from 2023, T2-FLAIR coronal, sagittal and axial images (middle and top left), and T1 axial image (bottom left), showing continuous layer of gray matter in antero-medial aspect of the right frontal lobe extending from the ventricular wall to cortex and causing bulge on the ventricular wall without intervening CSF cleft or white matter suggesting transmantle heterotopia. Colpocephaly and agenesis of the corpus callosum agenesis are also present.

The elective brain MRI performed in February 2023 (Fig. 1) revealed additional findings in addition to the known ACC. Indeed, transmantle heterotopia was found in the right frontal lobe, as well as pronounced atrophy of right precentral gyrus, colpocephalia, and smaller right cerebellar hemisphere compared to the normal left side. Given the overall atypical radiological picture, no specific radiological diagnosis was suggested.

Lumbar puncture revealed no cells, no signs of blood-brain barrier damage (normal albumin) or intrathecal antibody production, negative Borrelia serology, normal neurofilament, and normal neurodegeneration markers (Bamylo 40/20, beta-Amyloid 42, Fosfo-tau, and Tau protein).

A preliminary assessment made in a collegium of neurologists at our hospital suggested that malformations in an aging person, more pronounced in the right brain hemisphere, could explain the left-sided motor symptoms. However, the gait disturbance and pain in the lower extremities could also have a polyneuropathic component. For this reason, a neurophysiological examination was performed and revealed decreased amplitudes in the left nervus tibialis but no sign of peripheral neuropathy.

A follow-up visit 6 months later revealed substantial progress in hypomimia, hypokinesia, and a clearly identifiable gear phenomenon in the right arm and right leg. Therefore, a PET-CT exam was requested to confirm the suspicion of Parkinson´s disease.

**Fig. 2:**
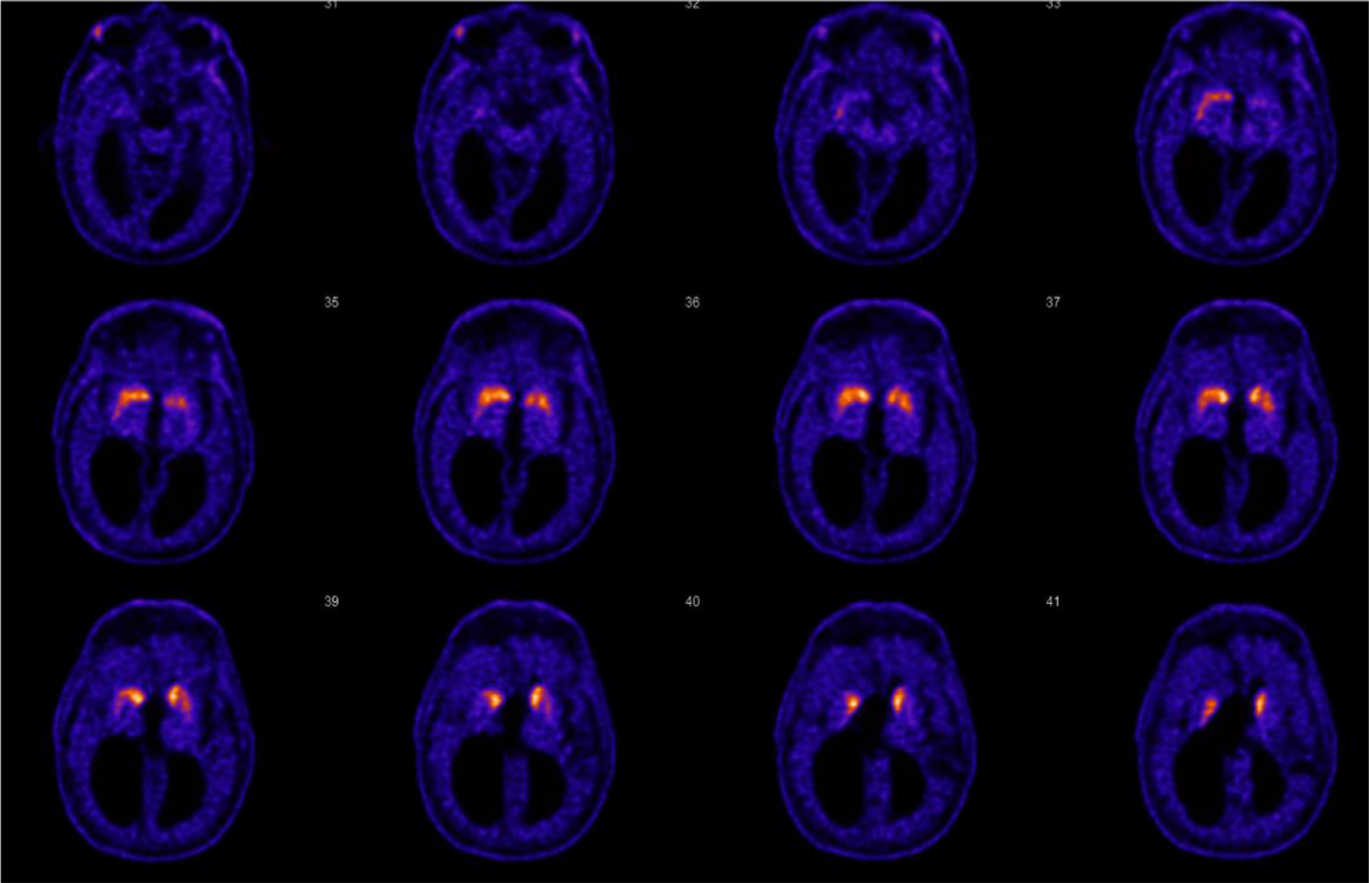
F18-FE-PE2I striatum brain PET-CT with signs of degeneration of the presynaptic nigrostriatal dopaminergic pathways were described, as in idiopathic Parkinson's disease or Parkinson-plus syndrome with left-sided predominance.

The F18-FE-PE2I striatum brain PET-CT (Fig. 2) revealed decreased uptake of the tracer bilaterally in the putamina and caudate nuclei, more pronounced on the left side. The scan confirmed signs of degeneration of the presynaptic nigrostriatal dopaminergic pathways, as in idiopathic Parkinson's disease or Parkinson-plus syndrome with left-sided predominance.

Based on the clinical and radiological image, therapy with Levodopa and dopamine agonist pramipexole was initiated, and the patient experienced an improvement in initiative and motor function but even some side effects such as minor hallucinations.

## Discussion and conclusion

ACC is characterized by complete or partial absent corpus callosum resulting from an insult during the gestation [1]. ACC may be isolated or associated with hundreds of other genetic anomalies, but also environmental factors, such as infections, maternal alcohol consumption or phenylketonuria [2,3]. ACC in children can be asymptomatic or manifest with intellectual disability, seizures [4], poor sleep quality [5], communication impairment [6].

In adults ACC has been linked to significant deficiencies in the conceptual, social, and practical aspects of adaptive behavior [7]. The relevance of ACC in the elderly is not defined but studies have shown that callosal tissue loss is implicated in the development of global cognitive as well as motor impairment [8].

Radiologically, transmantle heterotopia is a rare MRI finding where the cerebral mantle extends from the cortical surface to the ventricular wall without intervening white matter or cerebrospinal fluid cleft [9]. This type of heterotopia can be asymptomatic or associated to seizure disorders, loss of motor skills, and intellectual disability [10].

Combination findings of corpus callosum agenesis and heterotopia have been described in different genetic abnormalities but their clinical relevance is yet not well defined [11].

In the case presented in this report, the patient conducted a normal and well-functioning life until his 60 s, when he started to develop gait disturbances and cognitive impairment and, finally, was diagnosed with Parkinson's disease. Interestingly, the brain malformations were more pronounced in the right brain and cerebellum hemispheres. The reduced fine motor ability in the left hand could be clinically correlated to the right-sided atrophy of the precentral gyrus although it had not been described in previous neurological examinations or noted by the patient himself.

The reduced balance and gait disturbance may have also been associated partly with the right cerebellum atrophy and back pain. During the initial clinical neurological examination in 2023, strong clinical suspicion of Parkinson's disease was posed. The anatomical malformations found by CT and MRI examinations arguably made it more difficult to exclude a role of these malformations as responsible to patient´s symptoms as differential diagnosis and pose the diagnosis of Parkinson's and contributed to the delay in Levodopa therapy.

A take-home message may be to follow the clinical presentation when deciding to begin treatment despite accidental anatomical findings. However, it is not possible to exclude a contribution of the ACC and the heterotopia in the clinical manifestation and these brain malformations possibly contributed to the relatively quick progression of symptoms despite normal dementia markers and may be relevant in the long-term prognosis and adaptive capability of the brain to a strained situation caused by diminished dopamine production.

## Patient consent

Complete written informed consent was obtained from the patient for the publication of this study and accompanying images.
